# RNN-based detection of IoT malware using diverse feature engineering methods

**DOI:** 10.1038/s41598-026-51074-0

**Published:** 2026-05-11

**Authors:** Mahmoud Khaled Abd-Ellah, Nayera A. Alsayed, Osama M. Elkomy, Walaa M. EL-Hady

**Affiliations:** 1 Faculty of Artificial Intelligence, Egyptian Russian University, Cairo, 11829 Egypt; 2 Department of Information Technology, Faculty of Computers and Informatics, Zagazig University, Zagazig, Egypt

**Keywords:** Deep learning, Malware detection, IoT security, Network traffic, Recurrent neural networks (RNNs), Feature extraction, Engineering, Mathematics and computing

## Abstract

The Internet of Things (IoT) has rapidly expanded, introducing critical security vulnerabilities due to increasingly sophisticated malware that traditional detection methods struggle to identify. To enhance malware detection in IoT environments, we developed a framework leveraging recurrent neural networks (RNNs) integrated with advanced preprocessing and multilevel feature engineering techniques, including label encoding, MinMax scaling, TF-IDF, bag-of-words, word2vec, and principal component analysis. We evaluated three distinct RNN architectures on the UNSW-NB15 dataset via stratified fivefold cross-validation, and the final performance was assessed on the independent official test set, achieving progressively improved performance, with the final model demonstrating near-optimal classification results across accuracy, precision, recall, F1 score, specificity, and AUC. The results highlight the potential of combining deep learning techniques with diverse feature engineering strategies for improving malware detection in IoT environments. The proposed framework provides a scalable and experimentally validated approach for enhancing IoT malware detection against evolving threats.

## Introduction

Malware is recognized as a major threat to the digital world, as highlighted by recent theoretical and practical research. Over time, various malware prevention methods have evolved to increase security^1^. In the context of the Internet of Things (IoT), malware poses significant security challenges, particularly in identifying previously unknown threats. This difficulty arises primarily for two reasons: first, IoT devices often have limited computational and power resources, making the implementation of robust security measures challenging; second, the adoption of new network connectivity methods, such as cloud services, introduces additional security vulnerabilities, including susceptibility to malware attacks^2^.

Currently, cybersecurity initiatives utilize various scanning technologies to detect suspicious or malicious activities, employing techniques such as dynamic analysis, statistical analysis, static analysis, and content analysis^3^. However, attackers continuously develop new types of malware specifically designed to evade detection by conventional antivirus software^4^. Malware refers to software intended to monitor computer activities, compromise systems, or collect sensitive information^4^.

Malware encompasses a wide range of malicious software that targets personal computers, mobile devices, networks, and other systems. Common types include Trojan horses, ransomware, viruses, worms, and rootkits, each designed for purposes such as stealing sensitive data, enabling remote code execution, or damaging the victim’s system^5,6^. Detecting malicious software is crucial for protecting organizations and users. While signature-based detection methods were initially used, they are ineffective against sophisticated or novel malware. Consequently, researchers have proposed more advanced techniques, including pattern verification, heuristic detection, and behavior-based detection. In recent years, machine learning and data mining approaches have become increasingly popular for enhancing malware detection as threats continue to evolve^5^.

Malware patterns are always changing and becoming more complicated, so machine learning and artificial intelligence have both been shown to be effective approaches for identifying and classifying malware. Various machine learning techniques have been appslied to identify malicious assaults^7^. The three most popular machine learning techniques used to improve detection accuracy and classification efficiency in an important machine learning application area: malware classification^8^ are KNN, DT, and RF. Hidden Markov models, support vector machines, convolutional neural networks, and random forests are among the algorithms used in methods for detecting and classifying malware^9^. However, as malware detection models advance, cybercriminals are becoming more careful, and machine learning strategies are outperforming them^10,11^.

Malware detection is a critical cybersecurity challenge with implications that extend beyond technical issues, affecting organizations legally, financially, and reputationally^12^. Recent studies have employed both machine learning (ML) and deep learning (DL) techniques for malware detection^13^. While some researchers have focused on ML-based approaches, others have adopted DL solutions to increase detection accuracy^14–16^. Deep learning algorithms are particularly valuable because they can extract complex features from raw data and adapt to emerging threats, making them indispensable for malware detection. By utilizing both static and dynamic data, models such as convolutional neural networks (CNNs) and long short-term memory (LSTM) networks provide deeper insights into malware behavior^17,18^. However, despite their strong detection capabilities, deep learning models, including RNN-based architectures, often operate as complex and less interpretable systems. This black box nature may limit analyst trust and hinder practical deployment in security-sensitive environments. Recent research on explainable artificial intelligence (XAI) aims to improve transparency and interpretability in AI-driven cybersecurity systems. Integrating XAI techniques represents a promising future direction for enhancing trust and accountability in IoT malware detection frameworks^19,20^.

Despite the widespread use of deep learning algorithms for malware detection, several notable shortcomings persist in the current literature. Many studies are limited by small datasets, which restricts the generalizability of their findings to real-world malware scenarios^21–24^. Additionally, some approaches fail to leverage deep learning architectures specifically designed to uncover hidden patterns in malicious behavior^1^. The diversity of IoT-based threats is often underrepresented, as certain studies do not consider the full range of malware variants^25^. Furthermore, inadequate preprocessing and suboptimal feature extraction strategies have led to poor results in several studies^26^.

To address these challenges, this study proposes a detection system based on recurrent neural networks (RNNs). The approach incorporates diverse preprocessing and feature extraction techniques, including normalization, TF-IDF, word2vec, PCA, and recursive feature elimination (RFE), to enhance data quality and representation. By evaluating various RNN configurations, the framework aims to improve detection performance and effectively adapt to complex and evolving malware patterns.

This paper is structured into six main sections. Section "Literature Review" surveys existing research in the field. Section "Proposed Method" explains the proposed framework and its components. The implementation environment is described in Section "Environment". Section "Results" reports and discusses the experimental results, and Section "Discussion" concludes the study.

## Literature review

The number of malware attacks and the increasing number of people using smart devices are creating security concerns as malware research techniques improve. The complexity of protecting IoT environments is a result of the vast array of connected devices, each with varying hardware, software, and network architectural capabilities^27^. A literature review is a thorough and essential examination of a topic of interest that highlights both established and undiscovered facets of the discipline. In addition to guiding research toward subjects that need more investigation and examination, it also aids in identifying contentious issues and grounds of conflict^28^. By compiling and debating various study findings and trends, it offers a thorough examination of earlier studies and studies pertaining to a specific idea or strategy, which helps to create a solid awareness foundation^29^

Globally, the IoT is growing quickly, yet security is still a problem. Since these devices frequently lack continuous monitoring, machine learning approaches have shown promise in identifying malware on them^30^. Owing to its capacity to analyze large volumes of data with many layers of architecture, deep learning techniques and methods have emerged as crucial components of enhancing the analysis of malware in IoT contexts, facilitating increased threat detection efficiency and accuracy^27^.

The objective of this section is to examine current research on the use of deep learning and machine learning methods for detecting malware. The main goal will be to analyze the many methods used in the research, such as classifier design, feature extraction, feature selection, and preprocessing. We will also analyze the benefits and drawbacks of each study, pointing out any gaps in our understanding and potential difficulties in this area.

In^21^, the authors created a CNN-DMA malware detection system that uses deep learning and image processing techniques. The model classifies malware by using CNNs and extracting texture features. They used the Malimg dataset, which has 9,339 grayscale malware samples. The model was able to find 99% of the malware, but its limitations included an imbalance between malware classes and a small image size. In^22^, researchers developed new methods to detect attacks by combining fuzzy neural networks with software-defined networking (SDN). The adaptive neuro-fuzzy inference system (ANFIS) improves the accuracy and flexibility of detection. The NSL-KDD dataset has information on phishing, memory usage, the SNR, error messages, server overload, and SSH alarms. The framework can find 83% of attacks, although it has some problems, such as a small testing environment and dataset scope.

In^31^ employed TF-IDF and one-hot encoding to analyze data and depict features inside NetFlow data. They employed autoencoder and isolation forest models to find strange things. The isolation forest model achieves a perfect recall rate and a precision rate of more than 80% on all of the test datasets. The deep autoencoder model also exhibited high accuracy. The study recognized the difficulties of training autoencoders on extensive datasets.

In^32^ , methods such as tokenization, stemming, and stopword removal were used to create a framework for detecting malware and software piracy. It analyzes color pictures from malware executable files via a deep convolutional neural network and the TFIDF and LogTF weighting techniques. The framework detected only malware from known families, had no way to identify unknown families, and detected malware and piracy with 97.46% accuracy.

In^33^, this work first converts APK files to color images, and then the DCNN model is utilized to extract image features and detect malware. The researcher used the IIOT dataset, namely, the Leopard Mobile dataset, which contains 14,733 malware samples and 2486 benign samples, and the approach achieved an accuracy of 97.81%. This work is limited by the small dataset size and the absence of comparative analysis with other deep learning models. In^34^, ELBA-IoT, an ensemble learning-based botnet detection algorithm, was developed to protect IoT networks. The input data were first normalized via Z score normalization. The correlation coefficient score (CCS) method was subsequently used to select the best features. The researchers used the N-BaIoT-2021 dataset, which was used to train and test the model and had traffic from both normal and infected IoT devices. The experimental results revealed that ELBA-IoT achieved an accuracy of 99.6%.

In^35^, the authors presented the MADP-IIME protocol for identifying malicious assaults within IoT networks. To find bad assaults in Internet of Things (IoT) networks, a machine learning method was built. It employs four methodologies: naive Bayes, logistic regression, artificial neural networks (ANNs), and random forest. The protocol involves encoding labels, engineering features, scaling features, and handling of missing data. The MADP-IIME method was tested on the 823.69 MB IoT network intrusion dataset and shown to be more accurate than other methods for detecting intrusions, with a detection rate of 99.50%. However, the results may not be broadly relevant because of the particular dataset utilized for the performance metrics.

In^36^, a hybrid deep learning model was developed by integrating a CNN and LSTM. In addition, they utilized two benchmark datasets that are available to the public, namely, NSL-Botnet and UNSW-NB15. Using the NSL-Botnet dataset, the hybrid CNN-LSTM model was able to obtain a high detection accuracy of 99.4%. The accuracy of the model was established to be 93% when it was examined via the UNSW-NB15 dataset.

In^37^, this study looked at an IoT application that used an ARM CPU. This processor uses a recurrent neural network (RNN) based on long short-term memory (LSTM) to find malware in an IoT network. The LSTM structure has three layers and performs better than the RF, NB, SVM, MLP, KDD, DT, and AdaBoost classifiers do. The researchers employed a dataset of 281 malware samples and 270 benign samples for an IoT application. Next, they test the trained model with 100 additional samples of IoT malware. The accuracy rate for finding things was as high as 98%.

In^38^, a hybrid deep learning model was developed by integrating a CNN and LSTM. The authors applied various DL architectures using the CICIoT 2023 dataset, but the hybrid CNN-LSTM model achieved the best accuracy and performance, with 99.23%. In recent research on the detection of malware on the IoT, a wide variety of detection models that make use of deep learning, hybrid preprocessing, and a variety of datasets have been demonstrated. The results of these experiments are summarized in Table 1, which also provides a comparison of the different types of features, model architectures, and performance measures.

**Table 1:** Summary of recent malware detection techniques in IoT environments.

RF	Method			Result %				Dataset	Limitation	Advantage
	Preprocessing	Feature Selection	Detection	Acc	F1-score	Per scion	Recall			
^21^	Image conversion from binary to grayscale	–	CNN-DMA	99	**–**	–	–	Malimg Dataset	Imbalance between malware classes and small image size	Strong against methods that hide things, such encryption and packaging
^22^	–	–	FNN and ANFIS	83	–	–	–	NSL-KDD	Small testing environment and dataset scope	Lightweight design with reduced impact on system latency
^31^	TF-IDF	One-Hot Encoding	Isolation Forest	–	**–**	80	100	NetFlow	Autoencoder training on large datasets can be challenging and time-consuming, often requiring significant computer power, such as GPUs or distributed computing systems	Improved Model Performance: High recall rates were achieved to prioritize malware sample identification
			Auto encoder	–	**–**	90	100			
^32^	Tokenization Stemming Stopword Removal	TFIDF LogTF	DCNN	97.46	97.44	97.43	97.46	Collected from Google Code Jam (GCJ) and Leopard Mobile	There is no mechanism to detect malware from unknown families; only malware from known families is detected	**–-**
^33^	–	DCNN	DCNN	97.81	95.13	95.16	95.10	IIOT dataset, namely, Leopard Mobile dataset	Very small dataset size and the absence of comparative analysis with other deep learning models	**–**
^34^	Z score normalization	Correlation Coefficient Score approach (CCS)	ELBA-IoT (Ensemble Learning)	99.6	97.7	98.4	97.1	N-BaIoT-2021	–	**–**
^35^	–	–	MADP-IIME	99.5	–	–	–	IoT Network Intrusion	Performance indicators are based on a certain set of data, thus the findings are not as useful as they may be	**–**
^36^	one-hot encoding	**–**	CNN + LSTM (Hybrid DL model)	99.4	–	–	–	NSL-Botnet	**–**	**–**
	min–max Scaler			93				UNSW-NB15		
^37^	–	**–**	LSTM-based RNN	98.18	–	–	–	dataset comprising 281 malware and 270 benign ware	**–**	**–**
^38^	Label Encoding	**–**	CNN-LSTM (Hybrid DL model)	99.23	99	–	–	CICIoT 2023	**–**	**–**
	Z score normalization									

The analysis presented in Table 1 illustrates a recurring trend in the literature: the adoption of singular DL models and limited preprocessing strategies. While individual efforts have yielded promising results, integrated and comparative frameworks remain scarce. To bridge this gap, our study proposes a unified evaluation approach that applies multiple deep learning architectures in conjunction with a robust feature engineering process tailored to the IoT malware detection domain.

## Proposed method

**Fig. 1:**
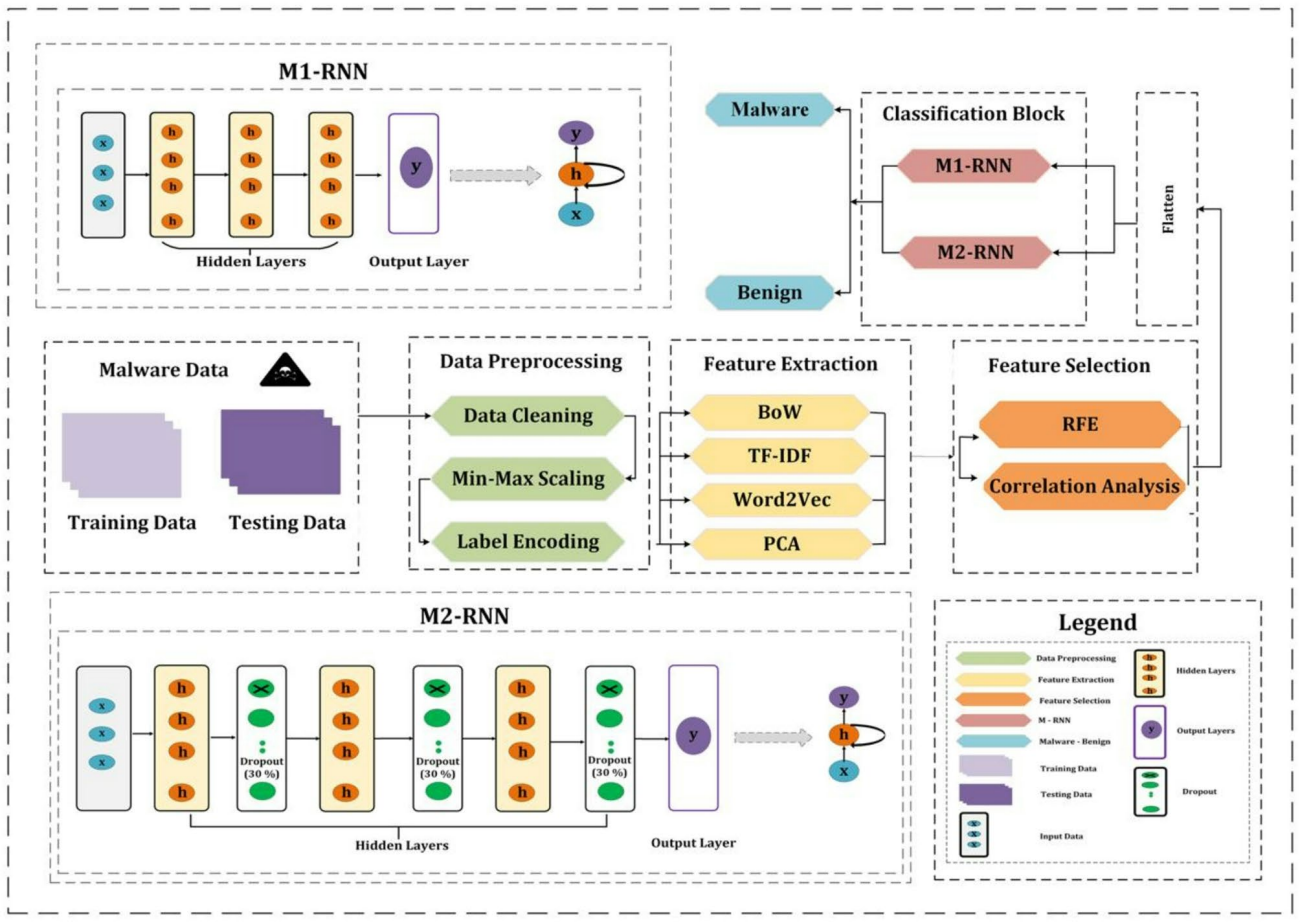
Proposed methodology framework for IoT malware detection via RNN models containing two M-RNN architectures.

The section devoted to the research methodology is shown here. It presents the complete experimental pipeline, including preprocessing, feature engineering, feature selection, and evaluation procedures used for IoT malware detection. The methodology was designed to ensure fair evaluation and reproducibility across all the proposed RNN-based models. Each stage of the pipeline, from data preparation to model evaluation, has been carefully planned to enhance detection performance and assess the efficacy of various architectures. Three experiments were conducted to prevent data leakage, and all preprocessing and feature extraction steps were performed exclusively on training partitions and then applied to validation and test partitions. Figure 1 shows that the suggested method includes preprocessing the data, extracting features, selecting features, and reshaping the input before training RNN models.

### Data Preprocessing

Preparing the dataset was a vital step before applying RNN models. Owing to the sensitive nature of sequential models, the raw input must be refined from noise, missing values, and irrelevant components. The UNSW-NB15 dataset underwent multiple preprocessing operations to ensure that the temporal information could be better represented and utilized by the RNN-based framework. To prevent data leakage, all preprocessing operations, including feature selection (RFE) and normalization (MinMaxScaler), were fitted exclusively on the training data. The learned parameters were then applied to the validation and independent test sets without refitting. This strict separation ensures that no information from the test set influences the model training process.

First, the training and testing sets were loaded into Pandas DataFrames. Because unprocessed network traffic data often cause problems such as extra records, values that are missing, and incorrect data entries, extensive preprocessing is necessary before training a model. First, all features are found to have missing values. When NaN values are present, machine learning models perform poorly. Although this facilitates analysis, it may lead to significant data loss, especially if missing data are prevalent. There may be bias in the results if the lost data are not random^39^. As a result, we removed the impacted rows. After that, duplicate entries are eliminated to prevent duplication and ensure that the model does not detect recurring patterns that can lead to bias. Additionally, to eliminate these values, some numerical variables are examined for negative values, such as dbytes (destination bytes), sbytes (source bytes), and dur (duration). Poor detection performance and inaccurate model training might result from aberrant or damaged data^40^.

#### Encoding of categorical features

Most of the methods used in deep learning and machine learning models require numerical input. Therefore, for the purpose of obtaining data that are ready for model training, categorical textual characteristics need to be converted into numerical values. For network traffic analysis, the dataset’s numerical feature set comprises a number of important metrics, including destination packets (dpkts), duration (dur), source bytes (sbytes), source packets (spkts), and destination bytes (dbytes). These attributes improve analysis and detection accuracy by characterizing the broad patterns of network traffic.

For deep learning models, categorical textual characteristics such as attack_cat (attack category), state (connection status), prototype (protocol), and service (network service) need to be encoded to be converted into numerical representations. All the distinct values in these category columns are analyzed to make this easier, and discrepancies such as unknown entries (“-”, “? “, and “unknown”) are found, changed to NaN, and then eliminated.

*Min–Max scaling *through the use of Scikit-learn’s MinMaxScaler, all the numerical characteristics were normalized to fall between 0 and 1. During training, this method reduces the impact of various feature scales, enhancing the consistency and convergence of models that contain deep learning technology^41^. Additionally, zeros were used to fill in missing values, guaranteeing the completeness of the data and the prevention of any possible discrepancies during the training of the model. In Equation (1), min–max scaling may be represented mathematically as follows^42^: The scaler parameters were fitted on the training subset only and then applied to the validation and test subsets to avoid data leakage.

1 $$k_{scaled} = \frac{{K - K_{\min } }}{{K_{\max } - K_{\min } }}$$

#### Label encoding

The process of label encoding, which entails providing an integer to each of the categories starting from 0 to N−1^43^, is a popular technique for altering data in categories into a number format. To enable the models to handle these characteristics efficiently, we used the label encoding approach for categorical encoding, which assigns each category a distinct number. Equation (2) provides the following mathematical expression for label encoding. Label encoders were fitted on the training data and subsequently used to transform the validation and test sets.

2 $$B = \left( {X_{i} } \right)$$

where:

- *x*_*i*_ is the categorical feature’s i-th element.
- B is the integer assigned to this category, where B belongs to the set of integers {0,1,…,n-1}, and n is the total number of distinct categories.

The use of these preprocessing approaches ensures that the input data are unambiguous, consistent, and suitably scaled. This, in turn, improves the model’s ability to recognize patterns, reduces the likelihood of overfitting, and enhances accuracy and performance across the board.

### Feature extraction

Once the dataset is cleaned and preprocessed, the next key step is selecting and extracting the most useful features that can guide the model during training; this is particularly important when dealing with high-dimensional data such as network traffic logs, where not every variable contributes meaningfully to the prediction task^40^. Its main objective is to change unorganized data into understandable formats that highlight particularly important patterns and trends to model training. Efficient feature extraction lowers the computational cost, improves model performance, and prevents overfitting.

Several feature extraction methods were used in this work to extract textual and numerical characteristics from the UNSW-NB15 dataset. By removing the information that is most significant from the data, these methods attempt to make feature vectors that can be used with deep learning models. To process the textual content in the dataset, we utilized techniques that focus on both word frequency and semantic meaning. For example, BoW and TF-IDF were applied to highlight frequent and contextually relevant terms within network traffic records. On the other hand, Word2Vec was used to embed words into vectors that preserve meaning on the basis of surrounding terms. To manage the complexity of the numerical data, PCA was introduced to eliminate dimensions and maintain only particularly instructive characteristics for model input.

To define the data precisely, enabling RNN-based classification models to make use of them and increasing the overall detection accuracy of malware attacks in network traffic, these feature extraction techniques are vital.

#### Bag-of-words (BoW)

In this study, CountVectorizer from scikit-learn was used to implement a bag-of-words (BoW) model, which ignores grammar and word order while maintaining word multiplicity. Network traffic data are represented in a sparse matrix, with each row denoting a record of data and each column reflecting the frequency of occurrence of a specific word in that record. This straightforward technique creates a vector space with hundreds of dimensions by creating a vocabulary from a corpus of writings and keeping track of how many times a word appears in every document. Each category variable is represented as a vector in this area, bringing variables with comparable contexts closer together and enhancing textual data processing^44^.

Mathematically, let V = {V_1_, V_2_, V_3_... V_n_} represent the vocabulary extracted from the dataset. For any given document d, the BoW vector is defined as^45^:

3 $$d_{ \to } = \left[ {Freq \, (V_{1} ,d),Freq \, (V_{2} ,d),Freq \, (V_{3} ,d),...,Freq \, (V_{n} ,d)} \right]$$

where *Freq (V*_*i, d*_*)* denotes the frequency of the word *V*_*i*_ in document d

In this way, the text is shown as a sparse vector, with a vocabulary term in each dimension. and its frequency of occurrence in the document. Owing to this representation, the model may focus on the presence and frequency of important terms that may indicate harmful behavior in network data. The vocabulary was learned from the training subset and then applied to the validation and test subsets.

#### Term frequency-inverse document frequency (TF-IDF)

TF-IDF is an approach to feature extraction that shows the essential nature of every word inside specific network traffic records by converting textual data into numerical representations. Two fundamental principles form the basis of TF-IDF:

• Term frequency (TF): This metric reflects how often a term is used in a document.

• One measure of a word’s usefulness or rarity in a corpus of documents is its inverse document frequency (IDF).

As indicated by Equation (4) in^3^, we utilized TF-IDF to determine how often a categorical variable appeared in our corpus. This was the score for word I in document j. The TfidfVectorizer used the category textual features and pulled out the TF-IDF values.

4 $$TFIDF \, = \, TF_{(n,m)} * IDF_{(n)}$$

where

- IDF = Inverse Document Frequency
- TF = Term frequency
- n = variable that is a word or a list
- m = Document or corpus or a subset that contains only categorical variables.
  5 $$TF \left( {n,m} \right) = \frac{Term n frequency in document}{{Total words in document}}$$
  6 $$IDF \left( n \right) = log_{2} \left( {\frac{Total documents}{{document with term n }}} \right)$$

It is possible to generalize Equations (5-6) above into a single component. Equation (7) displays the real mathematical formula as follows:

7 $$W_{(n,m)} = TF \left( {n,m} \right) * \log \frac{N}{df\left( n \right) }$$

In this scenario, N represents the total number of documents, tf (n, m) represents the frequency of n’s appearance in m, and df (n) represents the number of documents that contain n.

TF-IDF weights were computed on the basis of the training subset only. This method enables the model to assign higher weights to important and unique phrases detected in network traffic records while decreasing the influence of repetitive and common words that may not actually imply anything. Consequently, TF-IDF improves the capacity of classification models to detect malicious activity and intrusions.

#### Word2vec

In this study, the Word2Vec technique was applied to convert text-based categorical fields into numerical vectors that capture the contextual meaning of words within network traffic data. This method works by learning the relationships between words on the basis of how often they appear near each other, allowing the model to understand similarities in meaning or function^46,47^. The implementation was performed via the Gensim library, which offers efficient tools for natural language processing^48^. We trained the model on the relevant text features via a vector dimension of 100, a context window size of 5, and a minimum word frequency of 1 to ensure that even rare terms were included in the learning process.

In the skip-gram model used by word2vec, the core principle is to enhance the model’s ability to predict nearby words given a center word. This is done by optimizing the average logarithmic probability across all context‒target pairs^49^, which is mathematically described in Equation (8). Word2Vec embeddings were trained using only the training subset to ensure unbiased evaluation.

8 $$\frac{1}{T} \mathop \sum \limits_{t = 1 }^{T} \mathop \sum \limits_{ - c \le j \le c, j \ne 0} \log P\left( {Wt + j\left| {Wt} \right.} \right)$$

where:

- T is the corpus’s total word count
- c is the size of the context window.
- P (W_t+j_ │W_t_) is the conditional probability of a context word given the target word

Equation (9) uses the Softmax function to determine this conditional probability.

9 $$p\left( {w_{o} \left| {w_{1} } \right.} \right) = \frac{{\exp \left( {\mathop v\limits^{\prime}_{{w_{o } }} v_{w1 } } \right)}}{{\mathop \sum \nolimits_{w = 1}^{w} \exp \left( {\mathop v\limits^{\prime}_{{w_{o } }} v_{w1 } } \right)}}$$

This mathematical method enhances the model’s understanding of the meaning inherent in textual characteristics by capturing the syntactic and semantic connections between words by guaranteeing that words with similar contexts are mapped to similar vector representations.

This study integrated various feature extraction approaches to create a more comprehensive representation of network traffic data. Each technique captures different parts of the category features. BoW shows how often each word appears, providing a simple statistical picture of how often tokens appear. TF-IDF improves this representation by giving more weight to terms in the dataset that are more discriminative. On the other hand, word2vec captures semantic linkages by putting tokens into a continuous vector space, which keeps the meaning of the words in context. The suggested approach seeks to diminish information loss and enhance feature diversity by amalgamating frequency-based, importance-weighted, and semantic representations. This hybrid strategy allows the RNN models to learn from different points of view of the same traffic record. This could make them more robust and able to generalize better than if they used only one way to describe the data.

#### Principal component analysis (PCA)

This study employed principal component analysis (PCA) to reduce the dimensionality of the feature space while preserving the most informative variance in the dataset. PCA transforms the original features into a new set of orthogonal principal components that capture the maximum variance in the data^50^. By projecting the data into a lower-dimensional subspace, PCA reduces computational complexity and mitigates the risk of overfitting while maintaining the essential structural information of the dataset^51^. After the numerical features were concatenated with the TF-IDF and word2vec representations, the original feature space consisted of 231 dimensions. PCA was then applied to reduce this space to 50 principal components. The selected 50 components retained approximately 99.54% of the cumulative variance in the original feature space, indicating that nearly all the informative variance was preserved despite a substantial dimensionality reduction of approximately 78%. The number of components was determined via variance retention analysis to achieve an effective trade-off between computational efficiency and discriminative capability for malware detection. To ensure unbiased evaluation and prevent data leakage, PCA was fitted exclusively on the training subset and subsequently applied to the validation and external test subsets.

### Feature selection

Following preprocessing, an essential stage is feature selection, which removes characteristics from the dataset that do not address or help resolve the problem^40^. The features are chosen via correlation analysis and feature importance techniques.

#### Recursive feature elimination (RFE)

One feature selection approach that gradually eliminates irrelevant characteristics is referred to as RFE^52^. RFE with a random forest classifier is employed in the current study. This method repeatedly eliminates the least significant information while keeping the most relevant information to help identify network attacks^53^. To ensure that only the characteristics necessary for detecting network attacks are retained, the top 20 traits are chosen after several cycles. The deep learning models are subsequently trained on the basis of these characteristics. Previous studies have shown that reducing the feature space to the most informative subset can significantly improve classification performance while reducing model complexity^54^. There were 35 features in the first set of features. The feature space was reduced from 35 to the 20 most useful features. The features that were selected were dur, state, spkts, dpkts, sbytes, dbytes, rate, sload, dload, dloss, sinpkt, dinpkt, sjit, djit, tcprtt, synack, ackdat, dmean, response_body_len, and attack_cat. RFE was applied to the training subset only. During cross-validation, feature selection was performed within each training fold and then applied to the corresponding validation fold. The selection of the top 20 characteristics was based on empirical validation trials. These results showed that keeping more than 20 features only slightly improved performance while making the model more complex. This choice resulted in an appropriate balance between making the model simple, fast, and accurate.

#### Correlation analysis

**Fig. 2:**
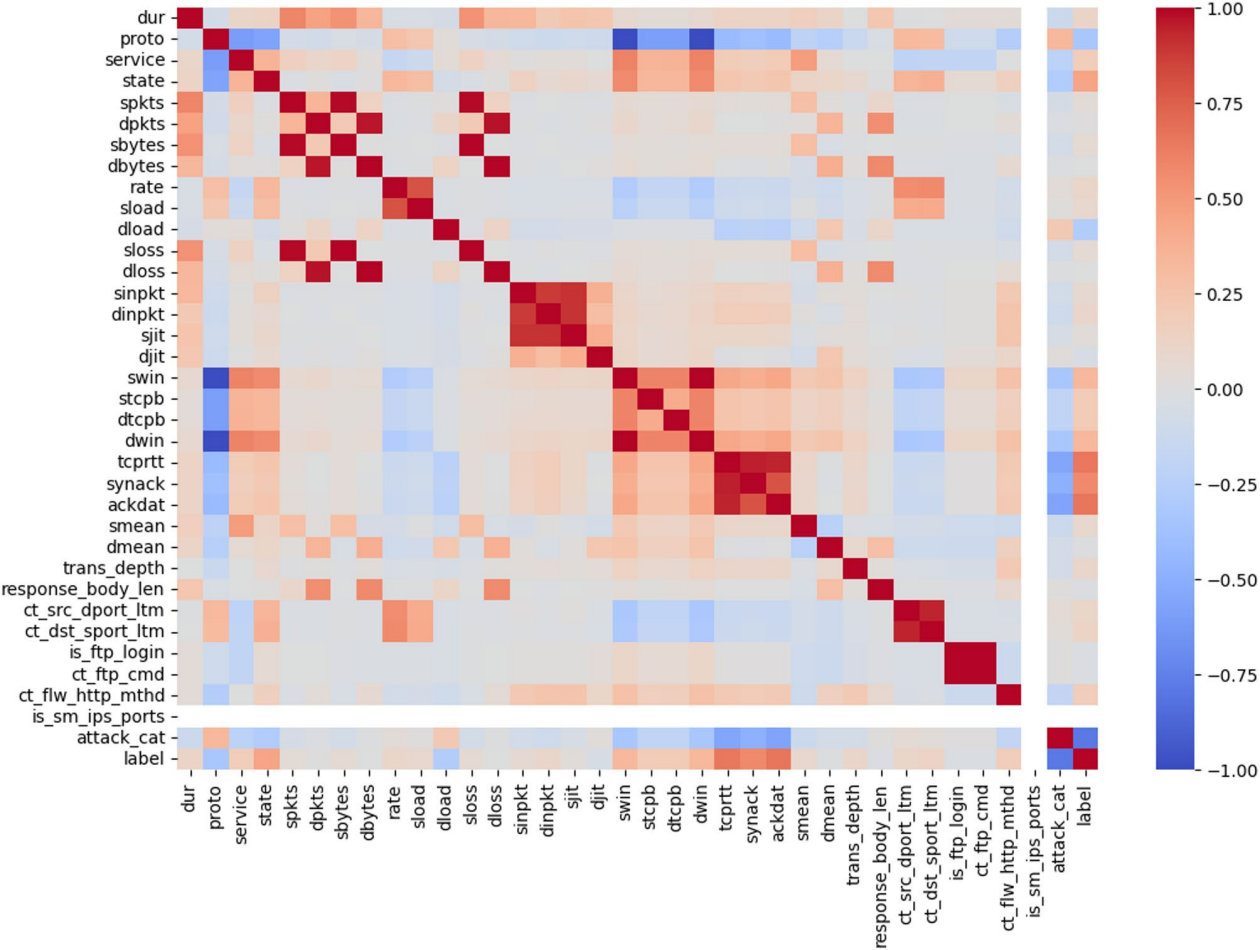
Correlation analysis of the dataset.

To examine the connections between various characteristics, correlation analysis is performed via heatmap visualization. The correlation matrix provides information about the correlations between the parameters, which facilitates the identification of any multicollinearity issues. The model’s capacity to generalize effectively may be impacted by duplication caused by features with extremely high correlations^40^. In this study, correlation analysis was used primarily as an exploratory step to visualize feature interactions and support the feature engineering process. The correlation heatmap is presented in Figure 2.

### Recurrent neural network (RNN) architectures

The ability of RNNs to process sequential data and identify temporal relationships between features makes them popular in deep learning applications. In this section, RNNs are used to detect malware traffic by utilizing their capacity to learn intricate patterns across temporally ordered data. Two RNN configurations were adopted (M1-RNN and M2-RNN) and evaluated across three feature engineering pipelines. Table 2 provides a comprehensive analysis of the architectural components and training configurations implemented in each model. The following section presents the evaluation metrics and detailed results.

**Table 2:** Comparative analysis of RNN architectures and training settings for the three RNN models.

Features	M1-RNN	M2-RNN
Input shape	(20,1)	(50, 1)
RNN layers (units)	3 × SimpleRNN (64 filters)	3 × SimpleRNN (64 filters)
Dense layers	Dense(1, sigmoid)	Dense(1, sigmoid)
Dropout	–	3
Loss function	BCELoss	BCELoss
Optimizer	Adam	Adam
Learning rate	0.001	0.0001
Epochs	10	15
Batch size	32	64

Although the UNSW-NB15 dataset does not represent long continuous temporal sequences, the ordered feature representations of network traffic records may exhibit contextual dependencies across attributes, and preservation of temporal ordering across samples was not required for RNN training. Recurrent neural networks (RNNs) have been adopted to explore whether sequential modeling of structured traffic features can enhance classification performance. Additionally, lightweight SimpleRNN layers were intentionally selected to evaluate the effectiveness of the reduced-complexity recurrent architectures in IoT malware detection scenarios.

#### M1-RNN

**Fig. 3:**
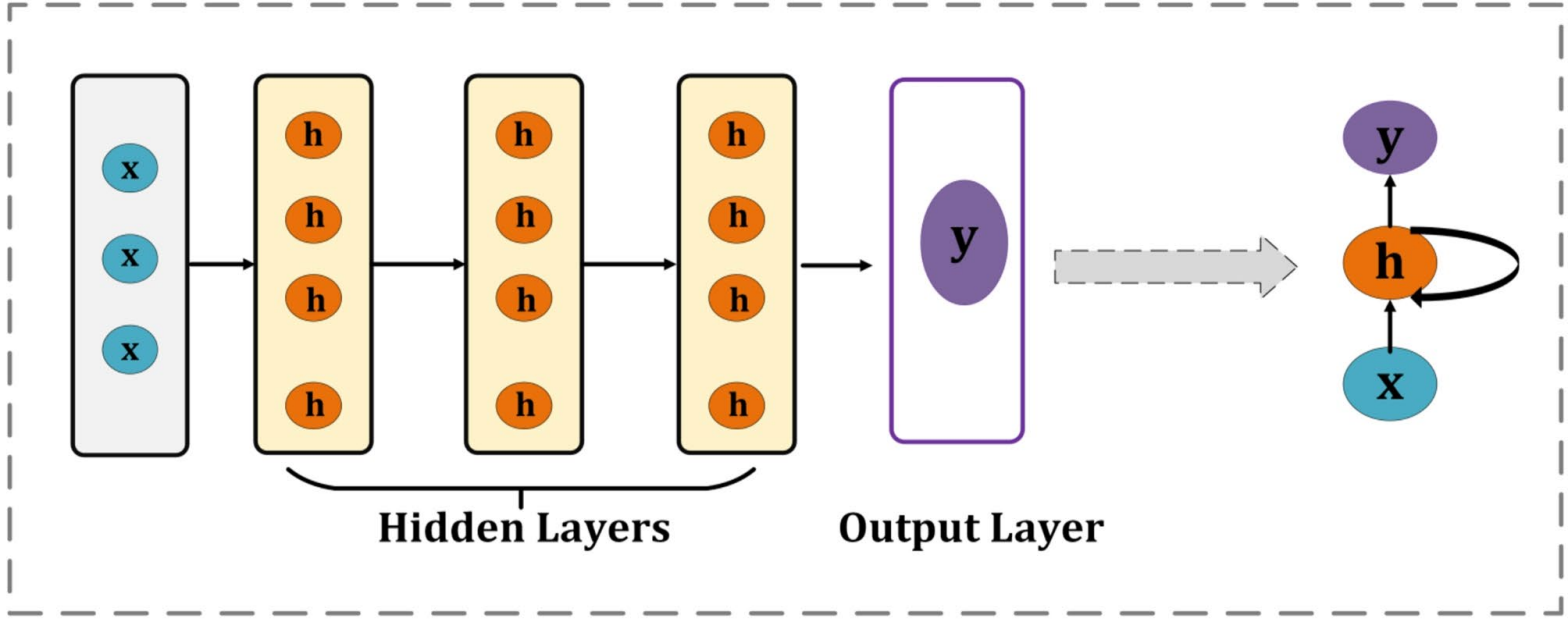
Architecture of the proposed M1-RNN.

In this study, an RNN was designed for the detection of malicious software by classifying network traffic into malicious and benign categories. The initial architecture that has been proposed includes three stacked SimpleRNN layers, each with 64 units, allowing the model to facilitate the efficient acquisition of patterns from consecutive inputs, and the detailed architecture is presented in Figure 3. We configured the first two SimpleRNN layers using return_sequences=True; that is, it returns the output of the entire sequence rather than just the final state. Therefore, the design ensures that deeper layers can process time-related information more efficiently while capturing long-term dependencies across input sequences. The first two SimpleRNN layers were configured with return_sequences=True, whereas the final recurrent layer used return_sequences = False to output a compact representation^55^. Then, a dense output layer with a sigmoid activation function was added to make binary classification easier. Finally, the output layer enables binary classification for malware detection by having a single neuron with a sigmoid activation function. For binary classification tasks in neural networks, the sigmoid activation function is often employed since it converts the values entered into a probability-like output ranging from 0 to 1, which aids in decision-making^56^. Sigmoid functions may be applied to deep learning, as shown by equation (10). More units allow the model to detect intricate sequence patterns, but too many units might result in overfitting^57^.

10 $$\Delta (x) = 1/(1 + e^{( - x)} )$$

#### M2-RNN

**Fig. 4:**
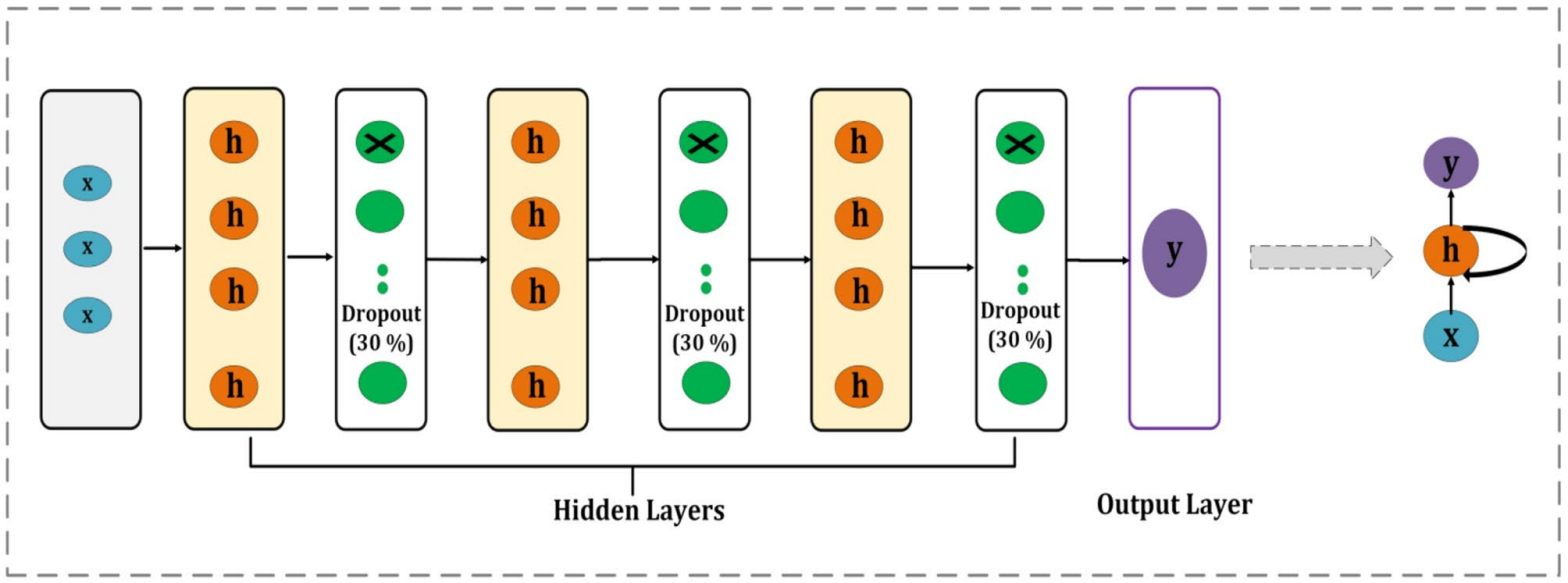
Architecture of the proposed M2-RNN.

The second proposed architecture is the same as the previous M1-RNN, but it introduces additional improvements, such as dropout layers, which are introduced in the M2-RNN, as shown in Figure 4. Each of the three-layered SimpleRNN layers in the improved RNN architecture has 64 units. Each recurrent layer was followed by a dropout layer with a rate of 0.3 to enhance generalization and lower overfitting. Return_sequences = True is set in the first RNN layer to guarantee that the sequence is sent to the following layer. For the purpose of carrying out binary classification, the final dense layer makes use of a sigmoid activation function.

### Proposed models

To investigate the influence of architectural complexity and optimization strategies on classification performance, three distinct model variants were developed. These models are named RFE-M1-RNN, BOW-M1-RNN, and PCA-M2-RNN. Each model introduces specific architectural adjustments intended to evaluate how different structural choices contribute to performance improvements. A detailed description of each architecture is provided in the following subsections.

In the RFE-M1-RNN, we cleaned the data by removing redundant entries, adding values that were missing, and correcting inconsistent data. This phase improved the dependability of training and evaluation by guaranteeing high-quality data and performance free of noise. Subsequently, label encoding was used to translate category characteristics into numerical values, ensuring their compatibility with deep learning models and preserving the implicit associations between text values. We used RFE to quantify each feature’s influence on the classification process to choose the most crucial features. Following feature selection and preprocessing, the features were then used to train on the M1-RNN architecture.

In BOW-M1-RNN, the representation of text network traffic data characteristics was improved by combining three feature extraction techniques: word2vec, TF-IDF, and BoW. The total feature space gained a unique feature from each procedure. TF-IDF improved upon BoW’s simple word frequency-based representation by assigning each word a significant weight on the basis of its uniqueness within the dataset. Word2Vec puts the meaning connections between terms into a dense vector space. After combining the features from all three methods into a single feature matrix, MinMax scaling was established to make the range of values more consistent and stop scaling problems. Finally, the features were used to train on the M1-RNN architecture.

In PCA-M2-RNN, initially, MinMaxScaler was used to normalize all numerical features, ensuring constant convergence throughout training by scaling them between 0 and 1. We used word2vec and TF-IDF for textual data. After PCA, the dimensionality decreased, which retained the most significant characteristics while removing extraneous information and increasing model generalizability and feature representation, as described in Section "Principal component analysis (PCA)". Finally, the features were used to train on the M2-RNN architecture.

### Training options

All the proposed models were trained via the Adam optimizer, which offers an excellent compromise between stability and convergence speed. M1-Block uses the Adam optimizer with a learning rate of 0.001, as the research in^58^ claims that since it ensures consistent updates free from excessive oscillations, many DL systems utilize the default value of 0.001, whereas M2-Block uses a learning rate of 0.0001. To maximize classification performance, we employed binary cross-entropy as the objective method in our design. This function is perfect for probabilistic models since it computes the error between the expected and real labels^59^. The binary cross-entropy loss (BCELoss) function is defined in^60^ and is shown in Equation (11).

11 $$BCELoss \, = \frac{1}{M}\mathop {\sum \left[ {(z_{i} \log \left( {p\left( {z_{i} } \right)} \right) + \left( {1 - z_{i} } \right) \log \left( {1 - p\left( {z_{i} } \right)} \right)} \right]}\limits_{i = 1}^{M}$$

where:

- M is the number of samples,
- ${z}_{i}$ is the actual class label (0 or 1),
- $\mathrm{p}\left({z}_{i}\right)$ is the predicted probability,
- $\mathrm{l}\mathrm{o}\mathrm{g}$ is the natural logarithm.

To prevent overfitting and allow for adequate learning, training was conducted for a limited number of epochs. If there are too few epochs, in some cases, the model could fail to learn complex structures, or if there are too many, it may become overfit and be unable to generalize the data for training^61^. For the initial experiments, an 80/20 stratified split was used. For rigorous evaluation, stratified 5-fold cross-validation was conducted, and the results are reported as the means ± standard deviations. The batch size was varied (32 or 64) depending on the design to balance computational efficiency and generalizability.

As shown in Table 2, both architectures share the same recurrent backbone composed of three stacked SimpleRNN layers with 64 hidden units. The primary difference lies in the regularization and training configuration. The M2-RNN architecture introduces dropout layers (rate = 0.3) after each recurrent layer and adopts a more conservative training configuration, including a smaller learning rate and slightly increased training epochs. These modifications aim to reduce overfitting and improve generalization, which is particularly important for intrusion detection tasks. In our experiments, the model configuration based on the M2-RNN demonstrated more stable learning behavior and superior overall performance compared with the baseline M1-RNN configuration. This suggests that the additional regularization and refined training settings contribute positively to model robustness. The hyperparameter configurations were determined through preliminary validation experiments and empirical evaluation. Different learning rates, batch sizes, and epoch settings were tested to ensure stable convergence and optimal validation performance. The final configurations were selected on the basis of a balance between classification accuracy and computational efficiency. Additionally, architectural settings such as the number of recurrent layers and units were aligned with commonly adopted practices in the deep learning-based intrusion detection literature.

## Environment

To ensure proper evaluation of our suggested models, we establish a testing environment by choosing a suitable dataset, developing data splitting methods, and utilizing common performance metrics. The previously discussed feature selection and dataset preparation techniques improved model performance. An outline of the dataset, data splitting technique and assessment measures utilized in this study are given in this section.

### Dataset

The UNSW-NB15 dataset was developed by UNSW Canberra’s Australian Centre for Cyber Security (ACCS) to evaluate intrusion detection systems (IDSs). This dataset includes nine different types of cyberattacks in addition to normal traffic^62^. It was created via the IXIA PerfectStorm program, which simulates both malicious and benign network traffic. At UNSW Canberra’s Cyber Range Lab, 100 gigabytes of raw traffic were captured and transformed into four million network flows^63^. With this dataset, we can identify nine distinct varieties of attacks, each of which represents a different type of malicious activity. Fuzzers attempt to disable programs by injecting unexpected inputs, whereas port scanning and other analysis techniques are used to gather network data. Backdoor attacks involve harmful malware that grants unwanted people access to a computer. Attacks known as DoS (denial of service) overwhelm network capacity to disrupt normal operations. To obtain unauthorized access, exploits seek weaknesses in systems. To circumvent security measures, generic attacks use encryption methods. In reconnaissance, network information is gathered by scanning and probing. Malicious code is immediately executed in system memory by shellcode assaults. Last, worms are malware that replicates itself and spreads over networks, damaging several computers without the user’s awareness or permission^62^.

In this study, the official predefined dataset partitions were strictly preserved. The dataset consists of 175,341 samples in the training set and 82,332 samples in the independent test set, and its distribution is presented in Table 3. This separation ensures that the final evaluation is conducted on unseen data, improving the reliability of the reported performance. Table 3 shows the class distributions in both the training and test sets. Although the dataset contains a greater proportion of malicious samples than benign samples, stratified validation and cross-validation were applied to preserve class proportions across all evaluation splits.

**Table 3:** Distribution in both the training and test sets.

		Dataset	Malicious	Benign	Total
Before preprocessing		Training set	119,341	56,000	175,341
		Test set	45,332	37,000	82,332
After preprocessing	RFE-M1-RNN	Training set	115,822	51,748	167,570
		Test set	7630	8978	16,608
	PCA-M2-RNN	Training set	119,341	56,000	175,341
		Test set	37,000	45,332	82,332
	BOW-M1-RNN	Training set	119,341	56,000	175,341
		Test set	37,000	45,332	82,332

For model development and hyperparameter tuning, the official training file was internally divided into 80% training and 20% validation subsets via stratified sampling to preserve the class distribution, as shown in Table 4. Stratified k-fold cross-validation was performed on the training dataset to further evaluate the robustness and generalizability of the proposed models. Additionally, 5-fold cross-validation was conducted on the training data to further assess robustness. Importantly, all preprocessing and feature engineering operations were fitted exclusively on the training subset and then applied to the validation and test subsets to prevent any potential data leakage. The independent test partition (82,332 samples) was strictly isolated and was not involved in any stage of model training, preprocessing fitting, or hyperparameter optimization.

**Table 4:** Dataset partitioning for fivefold cross validation.

Validation (35,068)	Train (35,068)	Train (35,068)	Train (35,068)	Train (35,069)	82,332	257,673
Train (35,068)	Validation (35,068)	Train (35,068)	Train (35,068)	Train (35,069)	82,332	257,673
Train (35,068)	Train (35,068)	Validation (35,068)	Train (35,068)	Train (35,069)	82,332	257,673
Train (35,068)	Train (35,068)	Train (35,068)	Validation (35,068)	Train (35,069)	82,332	257,673
Train (35,068)	Train (35,068)	Train (35,068)	Train (35,068)	Validation (35,069)	82,332	257,673

### Evaluation metrics

The trained RNN models were assessed by computing key performance metrics such as accuracy, precision, recall, F1 score, AUC and many measures, which were used to determine how well the model works. Additionally, the validation loss (val_loss) was recorded during training to determine how well the model converged and generalized. All of these evaluation metrics are derived from the four factors that can be found in the confusion matrix, which is displayed in Table 5. This matrix relies on the computed predicted class in relation to the actual class.

**Table 5:** Confusion matrix.

		Predicted class	
		Malware	Benign
Actual Class	Malware	True positive (TP)	False negative (FN)
	Benign	False positive (FP)	True negative (TN)

True positives, abbreviated as TP, indicate that the original data points and the predicted data points are both accurate; FP is an abbreviation for “false positives”. This indicates that the expected data points are accurate, but the original data points are incorrect. Both the initial data points and the predicted data points are incorrect, which is known as TN (true negatives), and FN is an abbreviation for “false negatives.” Therefore, when the predicted data points are incorrect, the real data points are accurate^64^. To further analyze the model’s classification performance, we computed the confusion matrix for our proposed models.

The performance of the models was assessed via multiple evaluation metrics derived from the confusion matrices, including accuracy (ACC), precision (PPV), recall (sensitivity/TPR), F1 score, specificity (TNR), negative predictive value (NPV), and area under the curve (AUC). The detailed values are presented in Table 5, and the corresponding ROC curves are shown in Figure 6.

The accuracy of the prediction is measured by adding together the two correct predictions (TP and TN) and then dividing by the entire number of datasets (P + N).

12 $${\text{ACC }} = \frac{{{\text{TP }} + {\text{ T}}N}}{P + N}$$

The precision (PREC) or positive predictive value (PPV) is measured by dividing the number of correct positive predictions (TPs) by the total number of positive predictions (TPs + FPs).

13 $${\text{PREC }} = \frac{TP}{{TP + FP}}$$

The recall or sensitivity or true positive rate (TPR) is measured by dividing the number of correct positive predictions (TPs) by the total number of positive predictions (P).

14 $${\text{REC }} = \frac{TP}{P}$$

The F1 score is another way to check how accurate the test is. Its precision and recall are used to compute it^65^.

15 $${\mathrm{F}} - {\mathrm{score}} = 2{*}\frac{{\text{Precision*Recall }}}{Precision + Recall}$$

The negative predictive value (NPV) is the probability that a sample is truly benign (nonattack) given that the model has classified it as benign^66^.

16 $${\mathrm{NPV}} = \frac{TN}{{TN + FN}}$$

The specificity or true negative rate (TNR) represents the proportion of benign (nonattack) samples that are correctly classified as benign out of all actual benign samples^66^.

17 $${\mathrm{TNR}} = \frac{TN}{{TN + FP}}$$

The area under the curve (AUC) is a statistical measure that indicates the probability that a classifier will provide a greater rating to a randomly picked positive observation than to a randomly selected negative observation, making it a popular evaluation metric for binary classification tasks^64^. The ROC curve is a receiver operating characteristic curve that plots metrics such as TP and FP to show how well a classifier performs at various threshold levels. The area under the ROC curve is calculated in two dimensions via the formula in equation (18) by the AUC-ROC, according to^64^.

18 $$AUROC \, = \mathop \smallint \limits_{0}^{1} \frac{TP}{P} d \left( \frac{FP}{N} \right)$$

## Results

### Performance evaluation

**Fig. 5:**
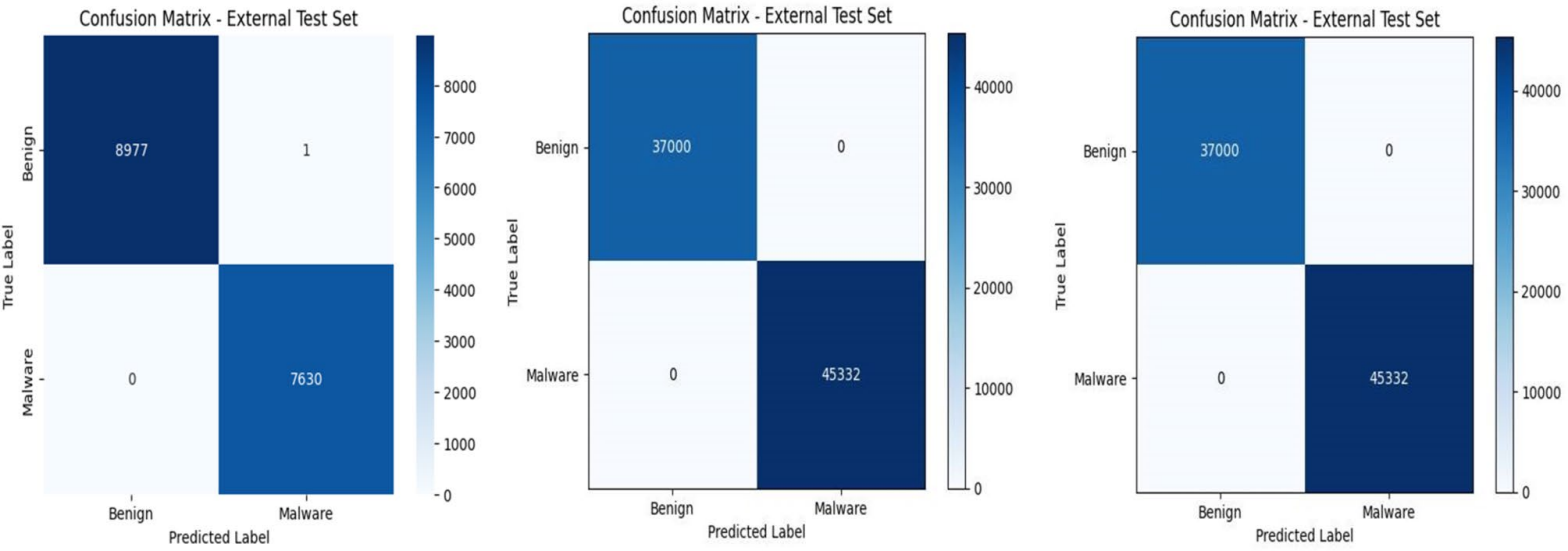
Confusion matrices of the proposed models: from left to right: RFE-M1-RNN, BOW-M1-RNN, and PCA-M2-RNN.

This section presents the performance evaluation of the proposed RNN-based models under different preprocessing and feature engineering configurations. The models were assessed in terms of accuracy, precision, recall, F1 score, and area under the ROC curve (AUC). The classification effectiveness was further examined via confusion matrices to analyze true positives (TPs), true negatives (TNs), false positives (FPs), and false negatives (FNs). Three RNN-based models (RFE-M1-RNN, BOW-M1-RNN, and PCA-M2-RNN) were evaluated on the UNSW-NB15 dataset, as shown in Table 6. The confusion matrices are illustrated in Figure 5 and provide detailed insight into classification behavior.

**Table 6:** Performance evaluation metrics of the proposed models.

RF	ACC	Precision (PPV)	Recall (Sensitivity/TPR)	F1 Score	Specificity (TNR)	NPV	AUC
RFE-M1-RNN	99.96 ± 0.0004	99.93 ± 0.0004	100 ± 0.0005	99.96 ± 0.0004	99.91 ± 0.0004	1 ± 0.0005	0.9996 ± 0.0004
BOW-M1-RNN	100 ± 0.0020	100 ± 0.0004	100 ± 0.0027	100 ± 0.0015	100 ± 0.0020	1 ± 0.0020	1 ± 0.0020
PCA-M2-RNN	100 ± 0.0000	100 ± 0.0000	100 ± 0.0000	100 ± 0.0000	100 ± 0.0000	1 ± 0.0000	1 ± 0.0000

Figure 5 shows the confusion matrices that show how well the three RNN models classify things. RFE-M1-RNN accurately classified most benign (8977) and malicious (7630) samples, with only one false positive and no false negatives. These results indicate strong detection capability with minimal misclassification. BOW-M1-RNN and PCA-M2-RNN demonstrated improved balance between classes and achieved zero misclassifications on the evaluated test set. While such results reflect excellent dataset-level performance, they should be interpreted within the context of specific dataset characteristics.

Overall, a progressive improvement trend is observed from Model 1 to Model 3, indicating that the applied feature engineering strategies and architectural refinements contributed positively to classification performance.

**Fig. 6:**
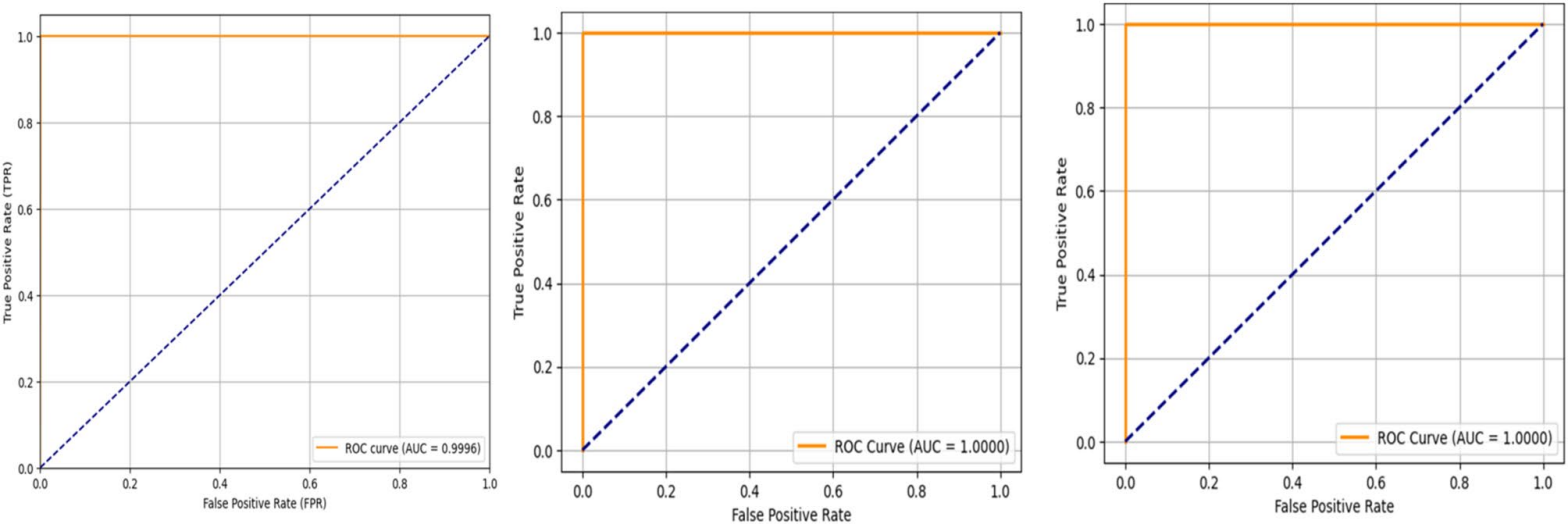
ROC curves of the proposed models: from left to right: RFE-M1-RNN, BOW-M1-RNN, and PCA-M2-RNN.

Table 6 summarizes the quantitative evaluation metrics, while Figures 5 and 6 present confusion matrices and ROC curves. All the models achieved consistently high performance, with RFE-M1-RNN reaching 99.96% accuracy, BOW-M1-RNN achieving 100% accuracy, and PCA-M2-RNN reaching 100% accuracy on the evaluated test set.

The final cross-validation performance of each model was the average of the five folds. This showed how well the model could predict and how stable it was. The detailed per-fold findings for all the RNN architecture means and standard deviations across folds are reported in Table 7. The proposed models show consistent performance across different folds, as seen. This shows that the models train in a steady way and are less sensitive to certain data partitions, which means that they work well with the supplied dataset. Importantly, cross-validation was only performed on the training set. The independent test set was retained for the final evaluation. This technique revents data leakage and guarantees an equitable evaluation of model generalizability. The current work does not include cross-dataset or real-world traffic evaluations because of limitations in dataset availability. However, the cross-validation analysis that was performed makes the experimental evaluation stronger by showing that it is stable over different training–validation splits (Tables 7 and 8).

**Table 7:** Stratified fivefold cross-validation results.

Method	Metrics	Flod-1	Flod-2	Flod-3	Flod-4	Flod-5	Average
RFE-M1-RNN	Accuracy	0.9997 ± 0.002	0.9996 ± 0.002	0.9994 ± 0.002	0.9999 ± 0.002	0.9988 ± 0.002	0.9994 ± 0.002
	F1-Score	0.9998 ± 0.0004	0.9996 ± 0.0004	0.9994 ± 0.0004	0.9999 ± 0.0004	0.9989 ± 0.0004	0.9995 ± 0.0004
	Precision	0.9995 ± 0.0004	0.9993 ± 0.0004	0.9988 ± 0.0004	0.9998 ± 0.0004	0.9991 ± 0.0004	0.9993 ± 0.0004
	Recall	1.000 ± 0.0005	1.000 ± 0.0005	1.000 ± 0.0005	1.000 ± 0.0005	0.9988 ± 0.0005	0.9997 ± 0.0005
BOW-M1-RNN	Accuracy	0.9991 ± 0.0020	0.9955 ± 0.0020	0.9995 ± 0.0020	0.9966 ± 0.0020	0.9999 ± 0.0020	0.9981 ± 0.0020
	F1-Score	0.9993 ± 0.0015	0.9967 ± 0.0015	0.9996 ± 0.0015	0.9795 ± 0.0015	1.000 ± 0.0015	0.9955 ± 0.0015
	Precision	0.9994 ± 0.0004	0.9991 ± 0.0004	0.9995 ± 0.0004	0.9999 ± 0.0004	1.000 ± 0.0004	0.9996 ± 0.0004
	Recall	0.9992 ± 0.0027	0.9943 ± 0.0027	0.9998 ± 0.0027	0.9951 ± 0.0027	0.9999 ± 0.0027	0.9976 ± 0.0027
PCA-M2-RNN	Accuracy	100 ± 0.0000	100 ± 0.0000	100 ± 0.0000	100 ± 0.0000	1 ± 0.0000	100 ± 0.0000
	F1-Score	100 ± 0.0000	100 ± 0.0000	100 ± 0.0000	100 ± 0.0000	1 ± 0.0000	100 ± 0.0000
	Precision	100 ± 0.0000	100 ± 0.0000	100 ± 0.0000	100 ± 0.0000	1 ± 0.0000	100 ± 0.0000
	Recall	100 ± 0.0000	100 ± 0.0000	100 ± 0.0000	100 ± 0.0000	1 ± 0.0000	100 ± 0.0000

**Table 8:** Comparative performance analysis of the proposed models against prior research.

RF	TP	FP	FN	TN	ACC	Precision (PPV)	Recall (Sensitivity/TPR)	F1 Score	Specificity (TNR)	NPV	AUC
^21^	–	–	–	–	99	95.8	98.9	–	–	–	98.9
^22^	–	–	–	–	83				–	–	–
^31^	–	–	–	–	–	90	100	95	–	–	0.99
^32^	0.9	0.01	0.1	0.99	97.46	97.43	97.46	97.44	–	–	–
^33^	0.99	0.068	0.014	0.93	97.81	95.16	95.1	95.13	–	–	–
^34^	–	–	–	–	99.6	98.4	97.1	97.7	–	–	–
^35^	–	–	–	–	99.5	–	–	–	–	–	–
^36^	–	–	–	–	93	–	94.7	–	–	–	0.95
^37^	–	–	–	–	98.18	–	–	–	–	–	–
^38^	–	–	–	–	99.23	–	–	99	–	–	0.9992
RFE-M1-RNN	8977	1	0	7630	99.96 ± 0.0004	99.93 ± 0.0004	100 ± 0.0005	99.96 ± 0.0004	99.91 ± 0.0004	100 ± 0.0005	99.96 ± 0.0004
BOW-M1-RNN	37,000	0	0	45,332	100 ± 0.0020	100 ± 0.0004	100 ± 0.0027	100 ± 0.0015	100 ± 0.0020	001 ± 0.0020	1 ± 0.0020
PCA-M2-RNN	37,000	0	0	45,332	100 ± 0.0000	100 ± 0.0000	100 ± 0.0000	100 ± 0.0000	100 ± 0.0000	100 ± 0.0000	1 ± 0.0000

The ROC analysis further confirmed the strong discriminative ability of the model. The AUC values for the three models were 0.9996, 0.9999, and 1.0000, respectively, indicating excellent class separability. As shown in Figure 6, the ROC curves remained close to the upper-left region, reflecting favorable trade-offs between sensitivity and specificity.

In summary, the results demonstrate that all three proposed models achieve highly competitive performance on the UNSW-NB15 dataset, with PCA-M2-RNN showing the best overall performance among the evaluated configurations.

### Computational complexity analysis

To evaluate the practical feasibility of the proposed architectures, a computational cost analysis was conducted. All three models share the same recurrent backbone and therefore contain an identical number of trainable parameters. Each model consists of 20,801 trainable parameters (81.25 KB), indicating a lightweight architecture suitable for deployment in resource-constrained IoT environments. The training time was recorded for each model under the same experimental setup in Google Colab. The RFE-M1-RNN model required approximately 189.52 s for training. The BOW-M1-RNN model required 4036.43 s, reflecting the increased computational overhead introduced by the combined high-dimensional text feature extraction techniques. The PCA-M2-RNN model required 2099.04 s, which represents a moderate increase compared with the baseline model. The inference time was measured for the best-performing architecture (PCA-M2-RNN), which yielded approximately 20.84 s on the validation set. Since inference involves only forward propagation without gradient updates, the runtime remains significantly lower than the training time. All runtime measurements were obtained via Google Colab under the available execution accelerator at runtime (GPU when available). Therefore, the reported values should be interpreted as indicative measurements reflecting relative computational overhead rather than strict hardware benchmarks. Overall, despite differences in preprocessing complexity, the models maintain a compact parameter footprint and manageable runtime, supporting their feasibility for practical IoT malware detection applications.

To provide a fair and consistent comparison with previous studies, Table 9 presents a comparison between the proposed framework and existing approaches that utilized the same UNSW-NB15 dataset. Since differences in datasets and preprocessing strategies may significantly influence reported performance metrics, focusing on studies that use the same benchmark dataset allows a more meaningful evaluation of the proposed method.

**Table 9:** Comparison with previous studies that used the UNSW-NB15 dataset.

RF	TP	FP	FN	TN	ACC	Precision (PPV)	Recall (Sensitivity/TPR)	F1 Score	Specificity (TNR)	NPV	AUC
^67^	–	–	–	**–**	97.67	97.72	97.68	97.7	–	–	–
^68^	85,728	87	208	46,413	99.67	99.62	99.78	99.7	–	–	–
^36^	–	–	–	–	93	–	94.7	–	–	–	0.95
^69^	–	–	–	–	92.4	–	–	–	–	–	–
^70^	–	–	–	–	94.44	92	100	96	–	–	–
RFE-M1-RNN	8977	1	0	7630	99.96 ± 0.0004	99.93 ± 0.0004	100 ± 0.0005	99.96 ± 0.0004	99.91 ± 0.0004	1 ± 0.0005	0.9996 ± 0.0004
BOW-M1-RNN	37,000	0	0	45,332	100 ± 0.0020	100 ± 0.0004	100 ± 0.0027	100 ± 0.0015	100 ± 0.0020	100 ± 0.002	1.0 ± 0.0020
PCA-M2-RNN	37,000	0	0	45,332	100 ± 0.0000	100 ± 0.0000	100 ± 0.0000	100 ± 0.0000	100 ± 0.0000	100 ± 0.00	1,0 ± 0.0000

Table 9 presents a comparison between the proposed framework and several previous studies that used the UNSW-NB15 dataset. While these studies report promising results, several limitations can be observed in their evaluation settings. For example, the study in^67^ reports good accuracy values; however, the evaluation is limited to a subset of performance metrics, which makes it difficult to assess the model’s detection capability fully. Similarly, the approach in^68^ achieves high performance but does not report all classification metrics, such as specificity and NPV, which limits the completeness of the evaluation. Other studies, such as^36^ and^69^, reported moderate performance levels, indicating that traditional machine learning or simplified deep learning architectures may struggle to fully capture complex network traffic patterns. The method presented in^70^ reports strong recall values; however, the overall evaluation remains limited to a small number of metrics and does not provide detailed stability analysis across multiple runs. In contrast, the proposed framework provides a more comprehensive evaluation by reporting multiple performance metrics, including accuracy, precision, recall, F1 score, specificity, NPV, and AUC. In addition, the results are reported as the means ± standard deviations across multiple runs, which provides a clearer indication of model stability. This more complete evaluation setup allows a more transparent assessment of the model’s performance on the UNSW-NB15 dataset.

## Discussion

Our RNN-based models consistently achieved state-of-the-art performance, as evidenced in the results section. The PCA-M2-RNN achieves perfect scores across all the evaluation metrics (100% ACC, precision, recall, F1 score, specificity, NPV, and AUC). The discussion in this section is based on the comparative results summarized in Table 8, which provides a comprehensive overview of both the raw confusion matrix values and the derived metrics (ACC, precision, recall, F1 score, specificity, NPV, and AUC). This comprehensive evaluation allows for transparent benchmarking against prior works.

### Limited evaluation metrics

Previous studies, such as^22,35^, and^37^, provided very limited information about the results. According to^22^, for example, their accuracy was only 83%, which is not only very low compared with our models (above 99.9%) but also incomplete because it does not include important metrics such as recall, precision, and specificity. Additionally^35^ achieved 99.5% accuracy but did not report recall, precision, or AUC, so robustness could not be checked. A previous study^37^ reported that the method was 98.18% accurate without any supporting measures, which makes it much less reliable. On the other hand, our work gives you a full set of data that come straight from the TP, FP, TN, and FN. This makes things clear and allows you to fully evaluate both false positives and false negatives.

### Weak recall and sensitivity

The recall (or sensitivity) metric is particularly crucial in malware detection, as false negatives can leave malicious traffic undetected. Prior studies such as^36^ reported recalls as low as 94.7%, and^33^ reported 95.1%, which means that their systems missed a nonnegligible portion of attacks. Similarly^31^achieved only 95% recall. These values may appear acceptable in general classification tasks but are dangerously low in IoT security contexts. Our models, by contrast, consistently achieved 100% recall, eliminating undetected threats and thus ensuring maximum system protection.

### Imbalanced or unstable results

A number of previous studies have shown that precision and recall are not balanced, which is directly related to problems with false positives and false negatives. For example^32^ reported 97.43% precision and a 97.46% memory rate, whereas^33^ reported a 95.16% precision rate and a 95.1% recall rate. When these gaps, even if they seem small, are actually very large problems: lower precision means more false positives (good traffic being mistakenly marked as bad), and lower recall means more false negatives (real attacks not being found). Both of these outcomes make it much less likely that malware will be found in IoT devices. Our RNN-based models, on the other hand, always gave almost equal and perfect values for precision and recall (up to 100%), which means that almost no false positives or negatives occurred. This balance ensures that the method is not only correct but also reliable and usable in the real world.

### Near-perfect but still Inferior

Some works, such as^21^ and^34^, have achieved strong performance but still fall short^21^ reported 99% accuracy and 98.9% recall, but this is still below our 100% performance, especially in terms of precision and AUC^34^ achieved 99.6% accuracy, which is good, but its recall was only 97.1%, making it vulnerable to missed malware. In contrast, our RNN models achieved both accuracy and recall scores of 100% on the evaluated dataset.

### AUC and overall robustness

The AUC values from only a few studies were given, such as^31^ with 0.99 and^38^ with 0.9992. Even though these are strong, they’re not perfect. Our models achieved an AUC of 1.0 on the evaluated dataset, indicating perfect discrimination between malware and normal traffic in this context.

Our work addresses all these gaps by presenting a transparent, balanced, and truly comprehensive evaluation. The proposed RNN models not only outperform existing methods in terms of accuracy but also achieve perfect recall, precision, and AUC—metrics that are critical for reliable malware detection in IoT environments. This establishes our approach as state-of-the-art on the evaluated dataset. In contrast to previous research, our evaluation clearly shows superior balance across all components of the confusion matrix. Specifically, our models achieved zero false positives (FPs) and zero false negatives (FNs) in multiple scenarios, implying that no innocuous data were misclassified as attacks and that no attacks were missed. This is a crucial advancement because previous studies with lower accuracies implied a larger rate of FP or FN, which is undesirable in security-sensitive IoT situations. Although the model achieved extremely high performance on the UNSW-NB15 dataset, further validation on additional datasets is needed to assess real-world generalizability fully.

### Real-world deployment

In addition to achieving strong classification performance, the proposed framework was designed with practical deployment considerations in mind. The architecture relies on relatively lightweight SimpleRNN layers and contains only 20,801 trainable parameters, which keeps the computational overhead low compared with that of more complex deep learning architectures. The experimental results also show moderate training and inference times under the experimental environment, indicating that the framework can operate efficiently without requiring excessive computational resources. These characteristics make the proposed approach suitable for integration into practical IoT security monitoring systems, such as network intrusion detection systems (NIDSs), where timely detection and computational efficiency are essential. By combining efficient feature engineering techniques with a compact recurrent architecture, the proposed framework provides a balanced trade-off between detection performance and computational cost, which supports its potential applicability in real-world IoT security environments.

### Explainability considerations

While the proposed RNN-based models achieved strong detection performance, deep learning approaches—including recurrent neural networks—are often considered complex and less interpretable systems. This “black-box” nature may limit analyst trust in security-sensitive environments such as IoT malware detection. Recent research on explainable artificial intelligence (XAI) aims to improve transparency and interpretability in AI-driven cybersecurity systems by providing insights into model decisions and feature importance^19,20^. In the context of sequential models, XAI techniques have been proposed to interpret the predictions of recurrent neural networks by identifying the contributions of input features and temporal dependencies that influence classification outcomes^71^. Methods such as SHAP-based explainability frameworks can highlight which network traffic attributes play a critical role in the decision-making process of RNN models, thereby helping analysts better understand model behavior and validate detection alerts. Recent studies have demonstrated that explainability techniques can effectively reveal feature contributions and temporal relationships in recurrent neural architectures, supporting the interpretability of deep learning models applied to security analytics^72^. Therefore, incorporating explainability considerations alongside performance evaluation represents an important step toward improving transparency and practical adoption of deep learning-based malware detection systems.

## Limitations and future work

Although the proposed framework achieved strong performance on the UNSW-NB15 dataset, several limitations should be acknowledged. First, the evaluation was conducted on a single publicly available dataset, which may not fully represent all real-world IoT network scenarios. Future studies should validate the proposed approach on additional benchmark datasets to further assess its generalizability. Second, the dataset represents processed network flow records rather than raw packet streams, which limits the assessment of real-time deployment performance. Third, lightweight SimpleRNN architectures were adopted in this study to balance performance and computational cost. While effective, more advanced recurrent or attention-based architectures could be explored in future research. Fourth, although stratified validation was applied, the dataset exhibited class imbalance, which may influence the performance metrics. Future work may investigate advanced imbalance-handling strategies and real-time adaptive detection mechanisms. Additionally, real-world IoT environments may involve zero-day threats and previously unseen attack patterns that are not fully captured in benchmark datasets^73^. Extending the proposed framework toward adaptive or continual learning approaches could improve resilience against emerging threats. Finally, deep learning models inherently operate as complex and less interpretable systems. The incorporation of explainable artificial intelligence (XAI) techniques such as SHAP or LIME represents an important future direction for enhancing transparency and analyst trust in IoT malware detection systems^74^.

## Conclusion

The proliferation of IoT devices has transformed the digital landscape but simultaneously introduced critical security vulnerabilities, with malware posing one of the most severe threats. Traditional detection methods have proven insufficient, necessitating advanced, adaptive solutions. This study addressed these challenges by developing a robust RNN-based framework enriched with diverse preprocessing and feature extraction techniques, including MinMax scaling, TF-IDF, Word2Vec, BoW, and PCA. Through three carefully designed experimental models, we demonstrated that the strategic combination of deep learning with advanced data representations not only enhances performance but also ensures balanced, comprehensive evaluation across all critical metrics. In particular, our RNN model achieves flawless results, reaching 100% accuracy, recall, precision, F1 score, specificity, and AUC. These outcomes establish our approach as a state-of-the-art solution, outperforming prior works that often suffer from limited evaluation metrics, imbalanced results, or weaker generalizability. Overall, the findings indicate that integrating deep learning with sophisticated feature engineering can enhance IoT malware detection performance on the evaluated dataset.

## Data Availability

The dataset in the manuscript is from public datasets. The data were sourced from two distinct datasets: https://www.kaggle.com/datasets/dhoogla/unswnb15?select=UNSW_NB15_training-set.parquet

## References

[1] M, G. & Sethuraman, S. C. A comprehensive survey on deep learning based malware detection techniques. *Comput. Sci. Rev.* **47**, 100529 (2023).

[2] Alrubayyi, H., Goteng, G., Jaber, M. & Kelly, J. Challenges of malware detection in the IoT and a review of artificial immune system approaches. *J. Sens. Actuator. Netw.* **10**, 61 (2021).

[3] Ram, P. S. Harsha, A. S. Shankari, E. U. and Rao, N. K. Detection of malware using signature based algorithm undergoing database verification.

[4] Gibert, D., Mateu, C., Planes, J. & Vicens, R. Using convolutional neural networks for classification of malware represented as images. *J. Comput. Virol. Hack. Tech.* **15**, 15–28 (2019).

[5] Aslan, Ö. A. & Samet, R. A comprehensive review on malware detection approaches. *IEEE Access* **8**, 6249–6271 (2020).

[6] Abijah Roseline, S. & Geetha, S. A comprehensive survey of tools and techniques mitigating computer and mobile malware attacks. *Comput. Electr. Eng.* **92**, 107143 (2021).

[7] Naseer, M. et al. Obfuscated malware detection and classification in network traffic leveraging hybrid large language models and synthetic data. *Sensors* **25**, 202 (2025). 39796992 10.3390/s25010202 PMC11723200

[8] Inayat, U., Zia, M. F., Mahmood, S., Khalid, H. M. & Benbouzid, M. Learning-based methods for cyber attacks detection in IoT systems: A survey on methods, analysis, and future prospects. *Electronics* **11**, 1502 (2022).

[9] Mehta, R., Jurečková, O. & Stamp, M. A natural language processing approach to malware classification. *J. Comput. Virol. Hack. Tech.* **20**, 173–184 (2024).

[10] N. G. Ambekar, N. N. Devi, S. Thokchom, and Yogita, TabLSTMNet: enhancing android malware classification through integrated attention and explainable AI,” *Microsystem Technologies,* vol. 31, pp. 695–713

[11] Ullah, F., Turab, A., Ullah, S., Cacciagrano, D. & Zhao, Y. Enhanced network intrusion detection system for Internet of Things security using multimodal big data representation with transfer learning and game theory. *Sensors* **24**, 4152 (2024). 39000931 10.3390/s24134152 PMC11243847

[12] Alomari, E. S. et al. Malware detection using deep learning and correlation-based feature selection. *Symmetry* **15**, 123 (2023).

[13] Vinayakumar, R., Alazab, M., Soman, K. P., Poornachandran, P. & Venkatraman, S. Robust intelligent malware detection using deep learning. *IEEE Access* **7**, 46717–46738 (2019).

[14] H. Darabian, S. Homayounoot, A. Dehghantanha, S. Hashemi, H. Karimipour, R. M. Parizi*, et al.*, “Detecting Cryptomining Malware: a Deep Learning Approach for Static and Dynamic Analysis,” *Journal of Grid Computing,* vol. 18, pp. 293–303, 2020/06/01 2020.

[15] Yazdinejad, A. et al. Cryptocurrency malware hunting: A deep recurrent neural network approach. *Appl. Soft Comput.* **96**, 106630 (2020).

[16] Jeon, S. & Moon, J. Malware-detection method with a convolutional recurrent neural network using opcode sequences. *Inf. Sci.* **535**, 1–15 (2020).

[17] Thakur, P., Kansal, V. & Rishiwal, V. Hybrid deep learning approach based on LSTM and CNN for malware detection. *Wirel. Pers. Commun.* **136**, 1879–1901 (2024).

[18] Akhtar, M. S. & Feng, T. Detection of malware by deep learning as CNN-LSTM machine learning techniques in real time. *Symmetry* **14**, 2308 (2022).

[19] I. H. Sarker, H. Janicke, A. Mohsin, A. Gill, and L. Maglaras, 2024 Explainable AI for cybersecurity automation, intelligence and trustworthiness in digital twin: Methods, taxonomy, challenges and prospects.” *ICT Express,* vol. 10, pp. 935–958

[20] 7 E. Kate and M. Song, “Explainable AI (XAI) in Cybersecurity: Bridging the Gap Between AI and Human Understanding,” 01/26 2025.

[21] Anand, A., Rani, S., Anand, D., Aljahdali, H. M. & Kerr, D. An efficient CNN-based deep learning model to detect malware attacks (CNN-DMA) in 5G-IoT healthcare applications. *Sensors* **21**, 6346 (2021). 34640666 10.3390/s21196346 PMC8512885

[22] F. Farhin, I. Sultana, N. Islam, M. S. Kaiser, M. S. Rahman, and M. Mahmud, “Attack Detection in Internet of Things using Software Defined Network and Fuzzy Neural Network,” in *2020 Joint 9th International Conference on Informatics, Electronics & Vision (ICIEV) and 2020 4th International Conference on Imaging, Vision & Pattern Recognition (icIVPR)*, 2020, pp. 1–6.

[23] El-Ghamry, A., Gaber, T., Mohammed, K. K. & Hassanien, A. E. Optimized and efficient image-based IoT malware detection method. *Electronics* **12**, 708 (2023).

[24] M. Shobana and S. Poonkuzhali, “A novel approach to detect IoT malware by system calls using Deep learning techniques,In: *2020 International Conference on Innovative Trends in Information Technology (ICITIIT)*, 2020, pp. 1–5.

[25] Safeer, E. Tahir, S. Nawaz, A. Humayun, M. Shaheen, M. and Khan, M. Advanced hybrid malware identification framework for the internet of Medical Things, driven by deep learning, *Secur. Privacy,* vol. n/a, p. e454, 2024/08/26 2024.

[26] Alkahtani, H. & Aldhyani, T. Botnet attack detection by using CNN-LSTM model for internet of things applications. *Secur. Commun. Netw.* **2021**, 340859 (2021).

[27] Qureshi, S. U. et al. Systematic review of deep learning solutions for malware detection and forensic analysis in IoT. *J. King Saud Univ. Comput. Inf. Sci.* **36**, 102164 (2024).

[28] Bolderston, A. Writing an effective literature review. *J. Med. Imaging Radiat. Sci.* **39**, 86–92 (2008). 31051808 10.1016/j.jmir.2008.04.009

[29] Paul, J. & Criado, A. R. The art of writing literature review: What do we know and what do we need to know?”. *Int. Bus. Rev.* **29**, 101717 (2020).

[30] B. Sharma, R. Kumar, A. Kumar, M. Chhabra, and S. Chaturvedi, “A Systematic Review of IoT Malware Detection using Machine Learning,” In: *2023 10th International Conference on Computing for Sustainable Global Development (INDIACom)*, 2023, pp. 91–96.

[31] Shi, T., McCann, R. A., Huang, Y., Wang, W. & Kong, J. Malware detection for Internet of Things using one-class classification. *Sensors* **24**, 4122 (2024). 39000901 10.3390/s24134122 PMC11243998

[32] Ullah, F. et al. Cyber security threats detection in Internet of Things using deep learning approach. *IEEE. Access* **7**, 124379–124389 (2019).

[33] Naeem, H. et al. Malware detection in industrial internet of things based on hybrid image visualization and deep learning model”. *Ad Hoc Netw.* **105**, 102154 (2020).

[34] Abu Al-Haija, Q. & Al-Dala’ien, M. ELBA-IoT: An ensemble learning model for botnet attack detection in IoT networks. *J. Sens. Actuator Netw.* **11**, 18 (2022).

[35] Pundir, S. et al. MADP-IIME: Malware attack detection protocol in IoT-enabled industrial multimedia environment using machine learning approach. *Multimed. Syst.* **29**, 1785–1797 (2023).

[36] Jain, S., Pawar, P. M. & Muthalagu, R. Hybrid intelligent intrusion detection system for internet of things. *Telemat. Inf. Rep.* **8**, 100030 (2022).

[37] HaddadPajouh, H., Dehghantanha, A., Khayami, R. & Choo, K. K. A deep recurrent neural network based approach for internet of things malware threat hunting. *Future Gener. Comput. Syst.* **85**, 88–96 (2018).

[38] Polat, O. et al. Temporal-spatial feature extraction in IoT-based SCADA system security: Hybrid CNN-LSTM and attention-based architectures for malware classification and attack detection. *IEEE. Access* **13**, 102109–102132 (2025).

[39] Palanivinayagam, A. & Damaševičius, R. Effective handling of missing values in datasets for classification using machine learning methods. *Information* **14**, 92 (2023).

[40] Imran, J. F. & Kim, D. An ensemble of prediction and learning mechanism for improving accuracy of anomaly detection in network intrusion environments. *Sustainability* **13**, 10057 (2021).

[41] Shone, N., Tran Nguyen, N., Vu Dinh, P. & Shi, Q. A deep learning approach to network intrusion detection. *IEEE Transac. Emerg. Top. Comput. Intell.* **2**, 41–50 (2018).

[42] El hairach, M. L., Tmiri, A. & Bellamine, I. Univariate outlier detection: Precision-driven algorithm for single-cluster scenarios. *Algorithms* **17**, 259 (2024).

[43] Wang, Y., Li, J., Yang, B., Song, D. & Zhou, L. Orthogonal matrix-autoencoder-based encoding method for unordered multi-categorical variables with application to neural network target prediction problems. *Appl. Sci.* **14**, 7466 (2024).

[44] Dahouda, M. K. & Joe, I. A deep-learned embedding technique for categorical features encoding. *IEEE. Access* **9**, 114381–114391 (2021).

[45] Kowsari, K. et al. Text classification algorithms: A survey. *Information* **10**, 150 (2019).

[46] Sun, J. et al. Categorizing malware via A Word2Vec-based temporal convolutional network scheme. *J. Cloud Comput.* **9**, 53 (2022).

[47] Wang, J. et al. LogEvent2vec: LogEvent-to-vector based anomaly detection for large-scale logs in Internet of Things. *Sensors* **20**, 2451 (2020). 32357404 10.3390/s20092451 PMC7249657

[48] N. Katricheva, A. Yaskevich, A. Lisitsina, T. Zhordaniya, A. Kutuzov, and E. Kuzmenko, “Vec2graph: A Python Library for Visualizing Word Embeddings as Graphs,” in *Analysis of Images, Social Networks and Texts*, Cham, 2020, pp. 190–198.

[49] Wang, X., Zhao, H. & Chen, H. Improved Skip-Gram based on graph structure information. *Sensors* **23**, 6527 (2023). 37514822 10.3390/s23146527 PMC10383593

[50] Abdi, H. & Williams, L. J. Principal component analysis. *Wiley Interdiscip. Rev. Comput. Stat.* **2**, 433–459 (2010).

[51] Kherif, F. & Latypova, A. Chapter 12 - Principal component analysis. In *Machine Learning* (eds Mechelli, A. & Vieira, S.) 209–225 (Academic Press, 2020).

[52] Priyatno A. and Widiyaningtyas, T. A systematic literature review: recursive feature elimination algorithms, *JITK (Jurnal Ilmu Pengetahuan dan Teknologi Komputer),* vol. 9, pp. 196–207, 02/01 2024.

[53] Yadav, N. S., Sharma, V. P., Reddy, D. S. D. & Mishra, S. An effective network intrusion detection system using recursive feature elimination technique. *Eng. Proc.* **59**, 99 (2023).

[54] Basha, S. J., Veeraiah, D. & Lingamgunta, S. AQU-IMF-RFE: An extended feature selection method for intrusion detection in IoMT data using aquila optimization-based mutual information and recursive feature elimination. *Discov. Internet of Things* **5**, 12 (2025).

[55] Elsayed, S., Mohamed, K. & Madkour, M. A. A comparative study of using deep learning algorithms in network intrusion detection. *IEEE Access* **12**, 58851–58870 (2024).

[56] Szandała, T. Review and comparison of commonly used activation functions for deep neural networks. In *Bioinspired Neurocomputing* (eds Bhoi, A. K. et al.) 203–224 (Springer Singapore, 2021).

[57] Ghosh, K. et al. The class imbalance problem in deep learning. *Mach. Learn.* **113**, 4845–4901 (2024).

[58] Gudla S. P. K. and Bhoi, S. K. A Study on Effect of Learning Rates Using Adam Optimizer in LSTM Deep Intelligent Model for Detection of DDoS Attack to Support Fog Based IoT Systems, In: *Computing, Communication and Learning*, Cham, 2022, pp. 27–38.

[59] Ruby, U. and Yendapalli, V. Binary cross entropy with deep learning technique for image classification, *Int. J. Adv. Trends Comput. Sci. Eng.,* 9(10) 2020.

[60] Guo, C., Chen, X., Chen, Y. & Yu, C. Multi-stage attentive network for motion deblurring via binary cross-entropy loss. *Entropy* **24**, 1414 (2022). 37420434 10.3390/e24101414 PMC9601862

[61] Li, H., Rajbahadur, G. K., Lin, D., Bezemer, C. P. & Jiang, Z. M. Keeping deep learning models in check: A history-based approach to mitigate overfitting. *IEEE Access* **12**, 70676–70689 (2024).

[62] Moustafa N. and Slay, J. UNSW-NB15: a comprehensive dataset for network intrusion detection systems (UNSW-NB15 network dataset), In: *2015 military communications and information systems conference (MilCIS)*, 2015, pp. 1–6.

[63] Moustafa, N. & Slay, J. The evaluation of network anomaly detection systems: Statistical analysis of the UNSW-NB15 dataset and the comparison with the KDD99 dataset. *Inf. Secur. J. Glob. Perspect.* **25**, 18–31 (2016).

[64] Saranya, T., Sridevi, S., Deisy, C., Chung, T. D. & Khan, M. A. Performance analysis of machine learning algorithms in intrusion detection system: A review. *Procedia Comput. Sci.* **171**, 1251–1260 (2020).

[65] Vujovic, Z. Classification Model Evaluation metrics. *Int. J. Adv. Comput. Sci. Appl.* **12**, 599–606 (2021).

[66] Berman, D. S., Buczak, A. L., Chavis, J. S. & Corbett, C. L. A survey of deep learning methods for cyber security. *Information* **10**, 122 (2019).

[67] Azeem, M., Khan, D., Iftikhar, S., Bawazeer, S. and Alzahrani, M, Analyzing and comparing the effectiveness of malware detection: A study of machine learning approaches, *Heliyon,* 10, 2024. 10.1016/j.heliyon.2023.e23574 PMC10770453 38187275

[68] Ali, S. et al. A novel approach of botnet detection using hybrid deep learning for enhancing security in IoT networks. *Alex. Eng. J.* **103**, 88–97 (2024).

[69] Al-Hawawreh, M., Moustafa, N. & Sitnikova, E. Identification of malicious activities in industrial internet of things based on deep learning models. *J. Inf. Secur. Appl.* **41**, 1–11 (2018).

[70] Ali, M. et al. Hybrid machine learning model for efficient botnet attack detection in IoT environment. *IEEE Access* **12**, 40682–40699 (2024).

[71] Akanksha P. and Manohar Naik S., “Deep Learning and Explainable AI: A Dual Approach to Network Security,” In: *Intelligent Computing and Communication*, Singapore, 2025, pp. 327–336.

[72] Saranya, A. & Subhashini, R. A. systematic review of explainable artificial intelligence models and applications: Recent developments and future trends”. *Decis. Anal. J.* **7**, 100230 (2023).

[73] Naeem, M. R. et al. Cyber security enhancements with reinforcement learning: A zero-day vulnerabilityu identification perspective. *PLoS ONE* **20**, e0324595 (2025). 40424227 10.1371/journal.pone.0324595 PMC12111634

[74] Farhan, M. et al. Explainable AI-driven security framework for cyber-physical production systems in Industry 4.0: Leveraging immersive embedded CIoT. *IEEE Trans. Consum. Electron.* **71**, 10446–10453 (2025).

